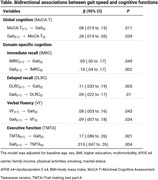# Bidirectional association between gait speed and cognition in community‐dwelling non‐demented older adults: A eight‐year longitudinal study

**DOI:** 10.1002/alz.087933

**Published:** 2025-01-09

**Authors:** Ying‐Hao Su, Jen‐Hau Chen, Yen‐Ching Chen, Jeng‐Min Chiou, Chenghsi Shiu

**Affiliations:** ^1^ Institute of Epidemiology and Preventive Medicine, College of Public Health, National Taiwan University, Taipei City, Taipei City Taiwan; ^2^ Department of Geriatrics and Gerontology, National Taiwan University Hospital, Taipei Taiwan; ^3^ National Taiwan University Hospital, taipei Taiwan; ^4^ Research Center for Genes, Environment and Human Health, National Taiwan University, Taipei Taiwan; ^5^ Institute of Epidemiology and Preventive Medicine, College of Public Health, National Taiwan University, Taipei Taiwan; ^6^ Institute of Epidemiology and Preventive Medicine, College of Public Health, National Taiwan University, No. 17, Xu‐Zhou Road, Taipei Taiwan; ^7^ National Taiwan University, Taipei Taiwan; ^8^ Department of Public Health, College of Public Health, National Taiwan University, No. 17, Xu‐Zhou Road, Taipei Taiwan; ^9^ Institute of Statistical Science, Academia Sinica, 128 Academia Road, Section 2, Nankang District, Taipei Taiwan; ^10^ Institute of Statistics and Data Science, National Taiwan University, Taipei, Taipei Taiwan; ^11^ Department of Social Work, National Taiwan University, Taipei City, Taiwan, Taipei City, Taipei City Taiwan

## Abstract

**Background:**

Slow gait speed and poor cognitions share numerous risk factors, including age, physical activities, chronic inflammation, education, metabolic abnormality, and the presence of multimorbidity. However, the causal relationship between gait and cognitions remains controversial. This study aimed to explore the reciprocal relationship of gait speed with global and domain‐specific cognition in non‐demented older adults.

**Method:**

This study used data from four waves of Taiwan Initiative for Geriatric Epidemiological Research (TIGER, 2013‐2021) with biennial assessments of casual walking speed and cognitions. Global cognition, memory, executive function, verbal fluency, and attention were assessed using Montreal Cognitive Assessment‐Taiwanese version (MoCA‐T), immediate and delayed recall, trail making test part‐A and part‐B (TMTA and TMTB), categorical naming task, and forward and backward digit span test, respectively. A cross‐lagged panel model (CLPM) and a linear mixed model were both used to investigate the bidirectional association between gait speed and cognitive function. Covariates included baseline age, sex, body mass index physical activities, higher education, physical activities, multimorbidity, smoking, Apolipoprotein E (APOE) ϵ4 status, family income, and marital status.

**Result:**

A total of 459 adults with a mean age of 74.5 (standard deviation 5.2) were included, and 239 participants (52%) were female. After adjustments for potential confounders, faster earlier gait speed was associated with better subsequent global cognition (MoCA‐T: β=.28, 95% confidence interval [CI]=.014‐.55), memory performance (immediate and delayed recall: β=.10, 95% CI=.04‐.17; β=.09, 95% CI=.02‐.17), verbal fluency (β=.09, 95% CI=.007‐.18), and executive function (TMTA: β=.15, 95% CI=.047‐.26). Similarly, better earlier cognitions of MoCA‐T (β=.08, 95% CI=.019‐.15), immediate and delayed recall (β=.09, 95% CI=.00‐.17; β=.11, 95% CI=.033‐.19), verbal fluency (β=.08, 95% CI=.003‐.16), and executive function (TMTA: β=.17, 95% CI=.086‐.26) were associated with faster subsequent gait speed. The results of linear mixed models were consistent with findings from CLPM analyses.

**Conclusion:**

We found reciprocal associations of gait speed with global cognition, memory, executive function, and verbal fluency. Early screening of gait speed and cognitive function helps identify individuals at risk and supports the maintenance of normal cognition and physical performance.